# Evaluation of late arterial acquisition and image quality after gadoxetate disodium injection using the CDT-VIBE sequence

**DOI:** 10.1038/s41598-022-15108-7

**Published:** 2022-07-06

**Authors:** Fen Liu, Feng Ma, Guanlan Zhou, Chongtu Yang, Bin Xiong

**Affiliations:** 1 Department of Radiology, The Central Hospital of Wuhan, Tongji Medical College, Huazhong University of Science and Technology, Wuhan, Hubei China; 2 Department of Otolaryngology, The Central Hospital of Wuhan, Tongji Medical College, Huazhong University of Science and Technology, Wuhan, Hubei China; 3 Department of Radiology, Union Hospital, Tongji Medical College, Huazhong University of Science and Technology, Jiefang Avenue #1277, Wuhan, 430022 China

**Keywords:** Cancer screening, Liver cancer

## Abstract

To explore the applicability of multi-arterial phase imaging technique in gadoxetate disodium-enhanced MRI. We studied 140 consecutive patients with suspected liver lesions who underwent gadoxetate disodium-enhanced MRI before surgery. All patients were randomized into three groups: group A (n = 50) was examined with VIBE-based single-artery phase imaging, group B (n = 44) with StarVIBE, and group C (n = 46) with CAIPIRINHA-Dixon-TWIST-VIBE (CDT-VIBE)-based multi-artery phase imaging. We evaluated the display rate of late arterial images and image quality in arterial phase images. We performed a study of 140 consecutive patients suspected with liver lesions who received gadoxetate disodium-enhanced MRI examination before surgery. All patients were randomly divided into three groups: group A (n = 50) was examined with single arterial phase imaging based on VIBE, group B (n = 44) was based on StarVIBE and group C (n = 46) was analyzed with multi-arterial phase imaging based on CAIPIRINHA-Dixon-TWIST-VIBE (CDT-VIBE). We evaluated the display rate of late arterial images and the image quality of dynamically enhanced images. Both radiologists had an almost perfect agreement (*Kappa* value > 0.8) in the assessment of late arterial and image quality. For late arterial acquisition, group C was superior to groups A and B (*x*^2^ = 18.940, *P* < 0.05); The image of phase 4 had the highest display rate in the late artery phase. For arterial phase image quality, there was no difference between groups A, B and C at five phases (*H* = 10.481, *P* = 0.106); and the best image quality score was lower in group C than in groups A and B (*H* = 8.573, *P* = 0.014).For the quality of the late arterial images, there was a statistical difference between the best images in groups A, B and C (*H* = 6.619, *P* = 0.037), and the images in group C were significantly better than those in group A (*P*_*.adj*_ < 0.05). By applying multi-arterial phase acquisition based on CDT-VIBE, gadoxetate disodium-enhanced MRI scanning can obtain a better late arterial phase and provide high-quality images with fewer motion artifacts.

## Introduction

Gadoxetate disodium is a dual gadolinium-based contrast agent for dynamic enhancement and specific hepatocyte imaging of the liver, which is advantageous for diagnosing patients with hepatic lesions^1,2^. However, some patients have had transient dyspnea or nausea after Gadoxetate disodium injection, causing transient severe motion (TSM)^3–5^ artifacts. TSM artifacts have harmful effects on the quality of images obtained during the arterial phase of dynamic contrast-enhanced (DCE) MRI imaging^3,4,6^, emphasizing the importance of reducing the interference of TSM artifacts during DCE MRI imaging with gadoxetate disodium^7^.

**Figure 1:**
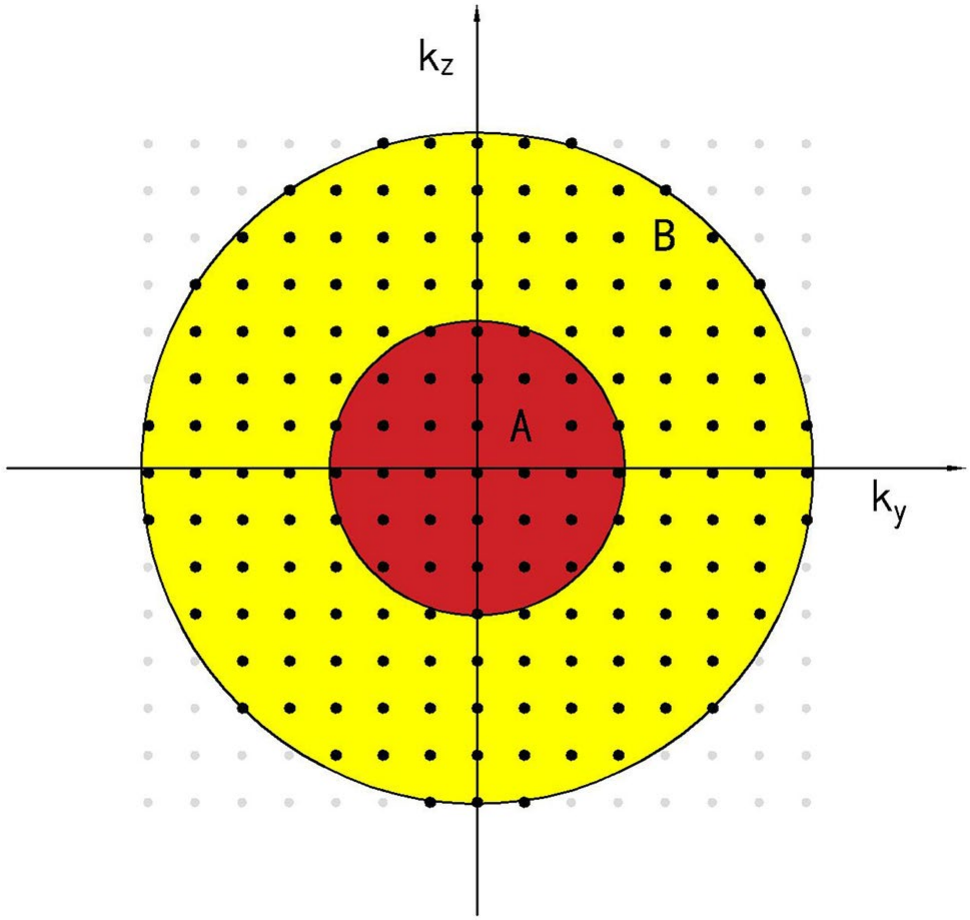
CDT-VIBE sequence k-space distribution.

StarVIBE (Star Volumetric Interpolated Breath-hold Examination) sequences based on radial acquisition in the k-space of gradient echoes^8–10^ can effectively reduce motion artifacts using the radial centre overlap filling method. It has good motion robustness based on the advantages of k-space centre oversampling and phase encoding with different readout directions. It can obtain good images in the free-breathing state^8,9^ and is widely used for imaging the gastrointestinal tract, liver, and fetus; this technique has the potential to reduce the interference of image TSM artifacts. The multi-artery phase acquisition CDT-VIBE uses time-resolved cross-random trajectory imaging. During k-space acquisition for 3D imaging, the A-zone phase space, which determines image contrast, is fully sampled. In contrast, the B-zone space is randomly undersampled outside the central region. By interleaving and reconstructing multiple phase-sampled signals before and after the B-region, which determines the image detail background, thus enabling fast scanning (Fig. 1), We can effectively reduce the transient severe motion artifacts caused by TSM^11–13^. Controlled aliasing in parallel imaging leads to higher acceleration (CAIPIRINHA) is a new acquisition scheme for volumetric imaging that modifies the image acquisition mode to exploit the sensitivity variation of the receiver coil array, thus improving the performance of parallel acquisition^11–13^. The image acquisition time for abdominal MRI can be further reduced using a higher acceleration factor. Time-resolved imaging with interleaved random trajectories (TWIST)^14,15^ is suitable for view-sharing techniques and can also be combined with VIBE sequences. The combination of view sharing and CAIPIRINHA techniques can further improve the temporal resolution while maintaining the spatial resolution of the acquisition and is theoretically suitable for dynamic enhanced imaging with temporary high resolution within a single breath-hold time. Uniform fat saturation is another primary requirement for successful dynamic abdomen imaging. The water-fat separation and fat compression technique (Dixon) is not sensitive to B0 unsaturation. The resulting CAIPIRINHA-Dixon-TWIST-VIBE sequence (CDT-VIBE) is a new technique for fast time-resolved dynamic 3D imaging of the abdomen with high spatial resolution. In this study, the acquisition speed of the CDT-VIBE sequence can be increased by five times compared with the initial sequence. After combining time-resolved cross-random trajectory imaging, we can obtain a set of images in 3 s. The spatial resolution can reach 0.7 × 0.7 mm^2^ after reconstruction using the difference algorithm. We can get five high-resolution images in one breath-hold for 15 s, providing at least one set of images with light or no artefacts.This is another potential technique to reduce the interference of TSM artifacts. Thus, this study aimed to compare gadoxetate disodium-enhanced MRI using a multi-arterial phase sequence based on CDT-VIBE with a single-arterial phase sequence based on VIBE and StarVIBE in the acquisition of late arterial and high-quality imaging.

CDT-VIBE multi-arterial phase sequence, single-arterial phase VIBE sequence, and StarVIBE sequence in contrast-enhanced magnetic resonance imaging of gadoxetate disodium liver.

## Materials and methods

The study protocol conformed to the ethical guidelines of the Declaration of Helsinki and was approved by the institutional review board of the Central Hospital of Wuhan. All patients signed an informed consent form before the MRI examination.

### Study population and data collection

**Figure 2:**
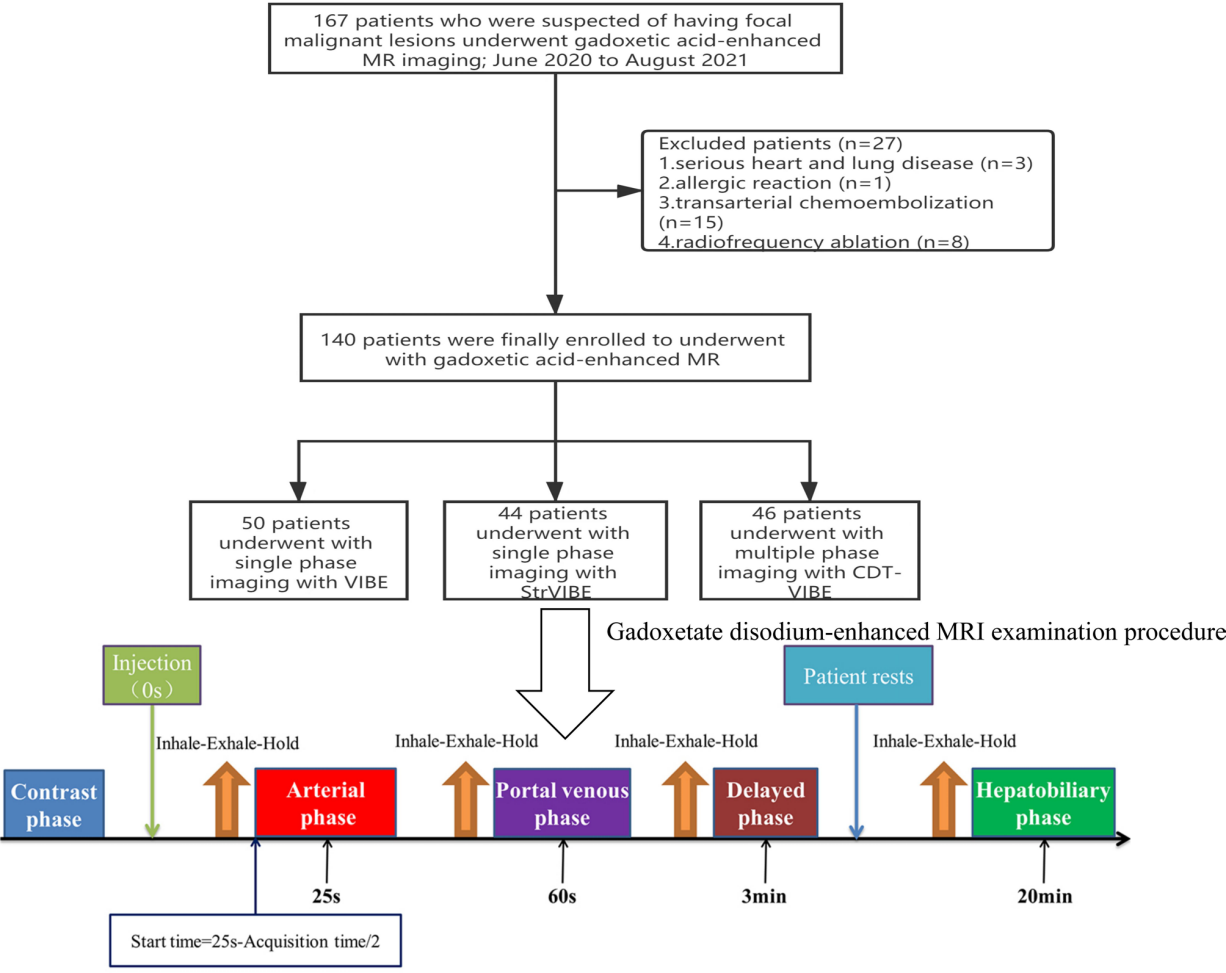
Patient inclusion and exclusion criteria and gadoxetate disodium-enhanced MRI examination procedure.

From June 2020 to August 2021, a total of 167 patients who were suspected of having liver lesions and underwent gadoxetate disodium-enhanced MRI examination were enrolled in this study (Fig. 2).

Inclusion criteria included:

1. Diagnosed as malignant focal hepatic lesions by B-ultrasound or CT.
2. Final diagnosis confirmed by pathology.
3. No contraindications of MRI examination.

Exclusion criteria included:

1. Suffering from severe cardiopulmonary diseases.
2. Allergic reactions during the examination.
3. Transcatheter arterial chemoembolization or radio-frequency ablation.

### MRI inspection equipment and imaging parameters

3.0 T magnetic resonance imaging system (Magnetom Skyra, Siemens Healthineers, Germany) was used for all patients' examinations, with an 18-channel phased-array body coil and 32-channel spinal coil. All the patients were randomly divided into three groups:

- Group A underwent single arterial phase imaging with VIBE sequence.
- Group B underwent single arterial phase imaging with StarVIBE sequence.
- Group C underwent multiple arterial phase imaging with the CDT-VIBE technique.

Gadoxetate disodium was used as a contrast agent. The dose was 0.025 mmol/kg body weight, and We injected the power at a rate of 2 ml/s. Each amount of gadoxetate disodium was followed by an intravenous saline chaser of equivalent volume to the injection. Before gadoxetate disodium injection, We performed the plain scan, followed by the arterial phase (25 s), portal vein phase (the 60 s), delay phase (3 min) and hepatobiliary phase (20 min) (Fig. 2). According to the standard scanning protocol, highly trained technicians or physicians complete the scanning process. The detailed parameters of the VIBE, StarVIBE and CDT-VIBE sequences are listed in Table 1.

**Table 1:** Three sets of sequence parameters.

Conventional	VIBE	StarVIBE	CDT-VIBE
Plane	Axial	Axial	Axial
TR/TE (ms)	3.31/1.3	2.83/1.48	3.97/1.23 and 2.48
Flip angle (°)	9	9	9
FOV (mm)	380	380	380
Spatial resolution (mm^2^)	1.2 × 1.2	1.2 × 1.2	1.2 × 1.2
Slice thickness (mm)	3	3	3
Slice gap (mm)	0.6	0.6	0.6
Number of slices	72	72	72
Fat saturation	SPAIR	SPAIR	Dixon
Bandwidth (khz)	450	820	1020
Acceleration factor	Phase 2/slice 2	/	Phase 1/slice 5
Acquisition time (s)	16	32	16
Motion compensation	Breath-Hold	Free Breath	Breath-Hold
Phases	1	1	5

### Image analysis

We used a Siemens Syngo-Imaging workstation to analyze the patient's MRI images. To ensure the objectivity of the blinded evaluation, two radiologists (with 6 and 13 years of abdominal diagnostic experience, respectively) evaluated the three sets of images, including a brief review of the arterial phase images and an assessment of image quality (including liver margin clarity, respiratory motion artefacts, and whether the images clearly showed the lesion). We resolved differences through discussion and consensus. The criteria of arterial phase score were as follows: 1 score = late arterial phase (hepatic artery and mild portal vein enhancement, no noticeable hepatic parenchyma enhancement or hepatic vein enhancement), 0 scores as no contrast medium in the hepatic artery, early arterial phase (just hepatic artery had contrast medium, but no enhancement of portal vein and hepatic parenchyma) and portal vein phase (enhancement of hepatic parenchyma and hepatic vein). Image quality includes four levels. (1) clear images without motion artefacts; (2) images with clearly discernible tissue structures and mild motion artefacts that do not affect the diagnosis; (3) images with poorly defined liver margins and lesions and moderate motion artefacts that can still be diagnosed; (4) images with obvious blurred liver margins and lesions that are poorly defined with severe motion artefacts that We cannot use for diagnosis.

### Statistical methods

We used SPSS 21.0 software for statistical analysis of the data. We assessed the consistency of image evaluation between two radiologists by calculating *kappa* values, in which 0.01–0.20 represents a slight agreement, 0.21–0.40 fair agreement, 0.41–0.60 moderate agreement, 0.61–0.80 good agreement, 0.81–0.99 almost perfect agreement and 1 perfect agreement. Categorical variables were expressed as the number of cases and percentages. *x*^2^ test was used to treat categorical variables. *Kolmogorov–Smirnov* test was used to test the normal distribution of the data, and normally distributed measures were expressed as mean ± standard deviation (*x* ± *s*). Nonparametric test Kruskal–Wallis was used to compare image motion within three sequences and five groups of multiple arterial periods. Differences in artifacts and further multi-period phase comparisons were performed for *α* with Bonferroni correction to reduce type I error. α was corrected according to the formula *α* = *α*′ × *m* (number of comparisons). *P* < 0.05 was considered a statistically significant difference.

### Ethics committee

The study protocol was approved by the institutional review board of the Central Hospital of Wuhan.

## Results

### Baseline characteristics

One hundred and forty patients, including 64 (45.7%) males and 76 (54.3%) females with a mean age of 58.5 ± 12.2 years, were included, and all eligible patients were randomized into three groups, with 50 in group A, 44 in group B, and 46 in group C (Fig. 2). Baseline characteristics did not significantly differ between three groups, including age (*F* = 0.241, *P* = 0.787), gender (*x*^2^ = 2.133, *P* = 0.344), body mass index (*x*^2^ = 0.384, *P* = 0.825), liver cirrhosis (*x*^2^ = 1.333, *P* = 0.514), ascites (*x*^2^ = 1.786, *P* = 0.842), and chronic obstructive disease (*x*^2^ = 1.830, *P* = 0.401). Both radiologists had an almost perfect agreement (*Kappa* value > 0.8) in the assessment of late arterial and image quality (Table 2).

**Table 2:** Consistency of scores between two physicians.

Group	Late arterial phase							Arterial phase image quality						
	A	B	C					A	B	C				
			1st	2nd	3rd	4th	5th			1st	2nd	3rd	4th	5th
*Kappa* values	0.958	0.906	0.877	0.910	0.912	0.942	0.928	0.946	0.936	0.966	0.933	0.931	0.933	0.901

### Comparison of late arterial capture rates

**Figure 3:**
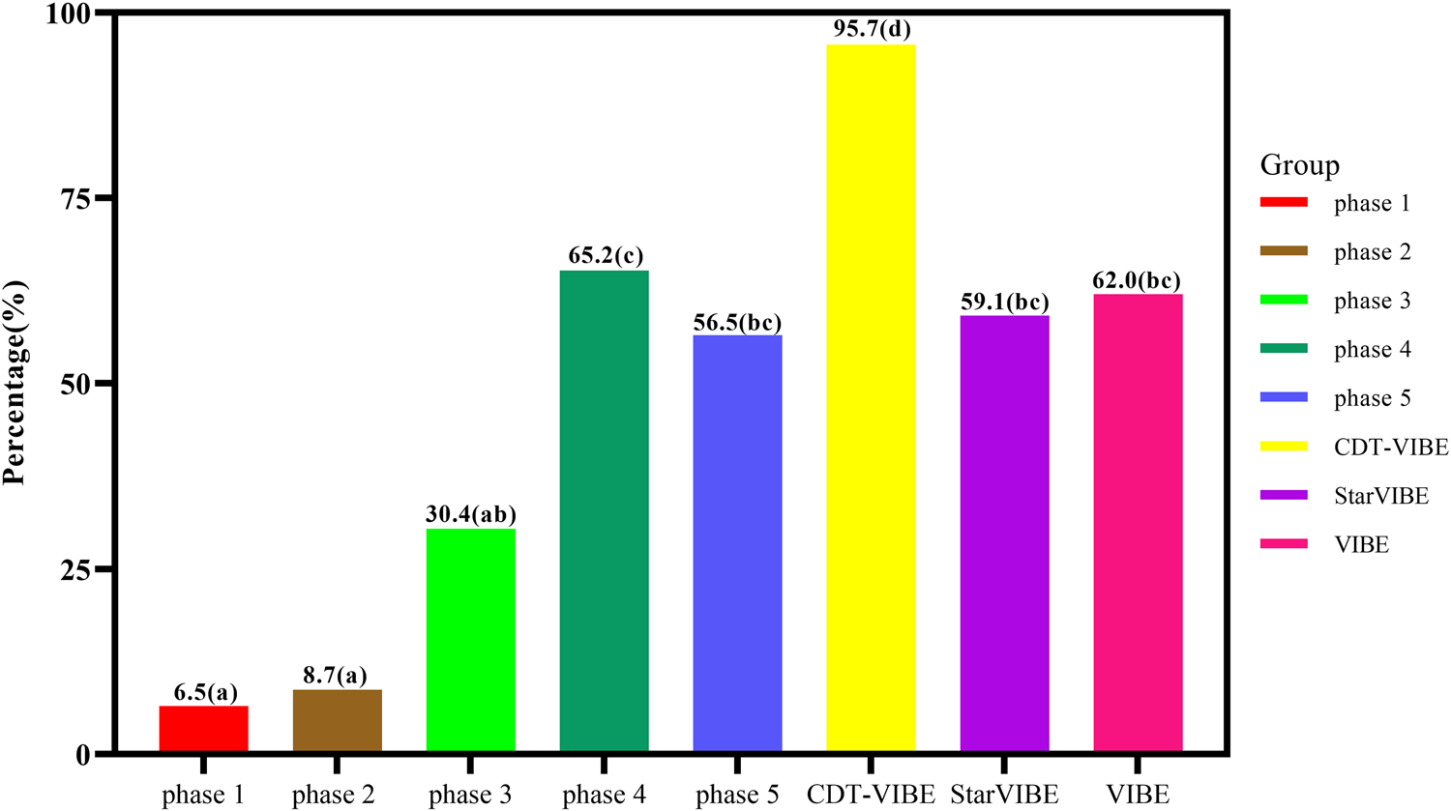
Late arterial capture rates.

The number of cases whose late artery was successfully observed was 31 in group A, 26 in group B, and 44 in group C, respectively, and the difference was statistically significant (*x*^2^ = 18.940, *P* < 0.05). There was no significant difference between group A and group B (*x*^2^ = 0.083, *P* = 0.773), whereas the difference between group A and group C (*x*^2^ = 15.876, *P*_*.adj*_ < 0.05) and group B and group C was significant (*x*^2^ = 17.393, *P*_*.adj*_ < 0.05). In group C, the display rates of late arterial phase 1, 2, 3, 4 and 5 in CDT-VIBE sequence were 6.5% (3/46), 8.7% (4/46), 30.4% (14/46), 65.2% (30/46), 56.5% (26/46), respectively, and the difference was statistically significant (*x*^2^ = 59.662, *P* < 0.05) . The image of phase 4 had the highest display rate in the late artery phase. The rate of late arterial capture was lower in the first two phases of the multi-arterial phase than in the last three phases and much higher in the last two phases (*P* < 0.05 for all), with images of the fourth phase showing the highest rate of late arterial capture, 34.8% higher than in the third phase (Table 3; Fig. 3).

**Table 3:** Distribution and percentage of arterial phase and image quality in three groups of images.

Score/case (%)	VIBE (50 Cases)	StarVIBE (44 Cases)	CDT-VIBE (46 cases)				
			Phase 1	Phase 2	Phase 3	Phase 4	Phase 5
**Late arterial phase**							
0	19 (38.0)	18 (40.9)	43 (95.3)	40 (87.0)	26 (56.5)	12 (26.1)	8 (17.4)
1	31 (62.0)	26 (59.1)	3 (6.5)	6 (13.0)	20 (43.5)	34 (73.9)	38 (82.6)
**Image quality**							
1	19 (38.0)	12 (27.3)	8 (17.4)	13 (28.3)	15 (32.6)	15 (32.6)	10 (21.7)
2	12 (24.0)	15 (34.1)	21 (45.7)	25 (54.3)	26 (56.5)	24 (52.2)	22 (47.8)
3	8 (16.0)	14 (31.8)	11 (23.9)	5 (10.9)	1 (2.2)	4 (8.7)	10 (21.7)
4	11 (22.0)	3 (6.8)	6 (13.0)	3 (6.5)	4 (8.7)	3 (6.5)	4 (8.7)

### Comparison of image quality

**Figure 4:**
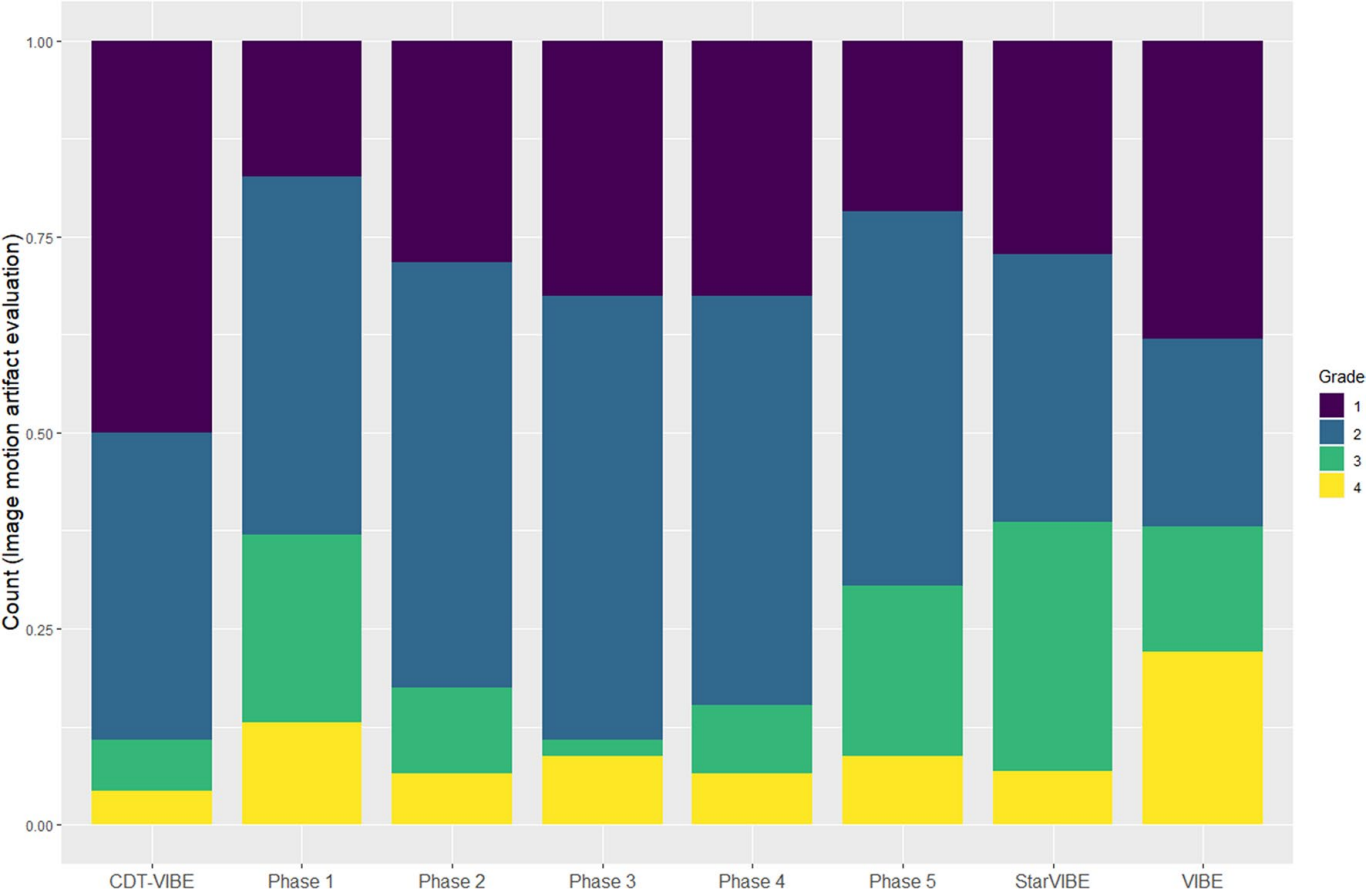
Evaluation of motion artifacts in multi-arterial CDT-VIBE sequence, single-arterial VIBE sequence and StarVIBE sequence images.

We set images with a image quality score of 1 or 2 as excellent images and images with a score of 3 or 4 as breath-holding failure images. The image with the lowest image quality score among the five-phase images of the multi-arterial CDT-VIBE sequence was the best CDT-VIBE image. There was no statistical difference between these seven groups of images in the arterial phase image quality score in groups A, B and C five phases (*H* = 10.481, *P* = 0.106); there was a statistical difference between the best image phases in groups A, B and C in the arterial phase image quality score (*H* = 8.573, *P* = 0.014). The best image phase scores in group C were better than those in groups A (*P*_*.adj*_ = 0.046) and B (*P*_*.adj*_ = 0.025), while there was no statistical difference in the image scores between groups A and B (*P*_*.adj*_ > 0.05). There were 31 cases in group A, 27 cases in group B, and 41 excellent images in group C. The difference was statistically significant (*x*^2^ = 11.225, *P* = 0.004). There was no significant difference between groups A and B (*P*_*.adj*_ > 0.05). Still, there were significant differences between groups A and C and between groups B and C (*P*_*.adj*_ < 0.05). 11 cases in group A, 3 cases in group B, and 2 cases in group C were observed to have severe motion artifacts (4 points). The difference was statistically significant (*x*^2^ = 8.722, *P* = 0.013). There was no significant difference (*P*_*.adj*_ > 0.05) between groups A and B and between groups B and C. At the same time, there was a significant difference (*P*_*.adj*_ < 0.05) between group A and group C and no significant difference (*P*_*.adj*_ > 0.05) (Table 3; Fig. 4).The quality of arterial late motion images (*H* = 6.619, *P* = 0.037) and the excellent rate (*x*^2^ = 11.649, *P* = 0.003) were statistically different between the best images of group A, group B and group C. There was a statistical difference between group A and group C (*P*_*.adj*_ < 0.05). At the same time, there was no statistical difference between group A and group B, group B and group C (*P*_*.adj*_ > 0.05 ).

## Discussion

In this study with 140 patients undergoing enhanced MRI, we found that the use of multi-arterial phase CDT-VIBE sequences captured late hepatic artery images promptly and with a higher probability than single-arterial phase VIBE sequences and StarVIBE sequences. Our study found that the CDT-VIBE multi-arterial phase sequence can obtain more arterial images than the other two single-arterial sequences through its high temporal resolution. In combination with the hepatobiliary phase images provided by the specific contrast agent gadoxetate disodium, it can display the contour and signal change information of liver lesions and achieve accurate localization and qualitative diagnosis of liver lesions.

Several studies^16–19^ have compared the capture rates of multi-arterial phase acquisition and conventional single arterial phase acquisition in the late arterial phase. In the survey carried out by Gruber et al.^17^. when using CDT-VIBE, the late hepatic artery phase was observed in 84.4% of the multiple arterial phases and 56.7% of the single arterial phase (*P* < 0.001). Wei et al.^18^ found that 95.4% of the multi-arterial phase was captured in the late arterial phase, while the capture rate of the single arterial phase was 73.1%(*P* < 0.001). Similarly, Our results showed that the late arterial phase capture rate of CDT-VIBE in the multi-arterial phase (95.7%) was much higher than that of VIBE (62.0%) and StarVIBE (59.1%) using single arterial phase (*P* < 0.05). However, In the previous studies^16,17^, only 2 or 3 arterial phases were used to collect multi-arterial phases to obtain a suitable late arterial period. In this study, five arterial periods were obtained with a time resolution of 3 s for each, so there will be more opportunities to observe the dynamic enhancement process of the lesions but not just the late arterial phase. Our results showed that the occurrence rate of the late artery was the highest in phase 4, the early phase of artery appeared in phase 1 or 2, and the early portal vein phase or portal vein phase appeared in phase 5. Therefore, by collecting the multi-arterial phase with 5 phases, we covered the enhancement process from the early arterial phase to the portal venous phase, which is of great significance for diagnosing of liver lesions^19^.

**Figure 5:**
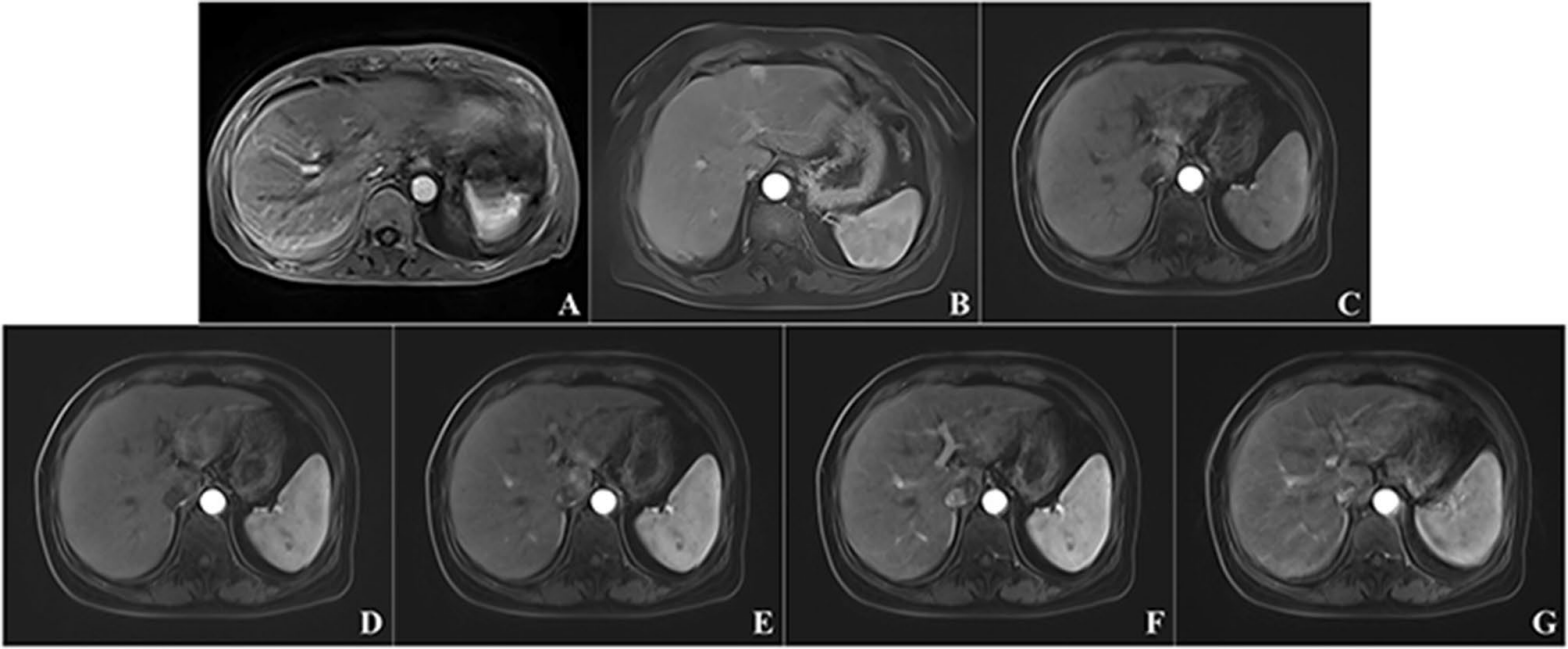
(**A**) shows the VIBE sequence image, and (**B**) shows the StarVIBE sequence image. (**C**)–(**G**) show the CDT-VIBE images. 1–5 phases of CDT-VIBE sequence, in which (**C**) and (**D**) show early arterial phase, only abdominal aorta and hepatic artery are enhanced; (**E**) Late arterial phase, with an enhancement of the abdominal aorta and hepatic artery and slightly enhancement of hepatic portal vein; (**F**) and (**G**) show the portal vein period, with portal vein enhancement and liver parenchyma enhancement. The scores of artefact degree showed that (**A**) was 4 points, (**B**) was 1 point, (**C**) was 2 points, (**D**)–(**F**) was 1 point, (**G**) was 4 points, and the motion artefact in the fifth phase was heavier than that in other phases.

**Figure 6:**
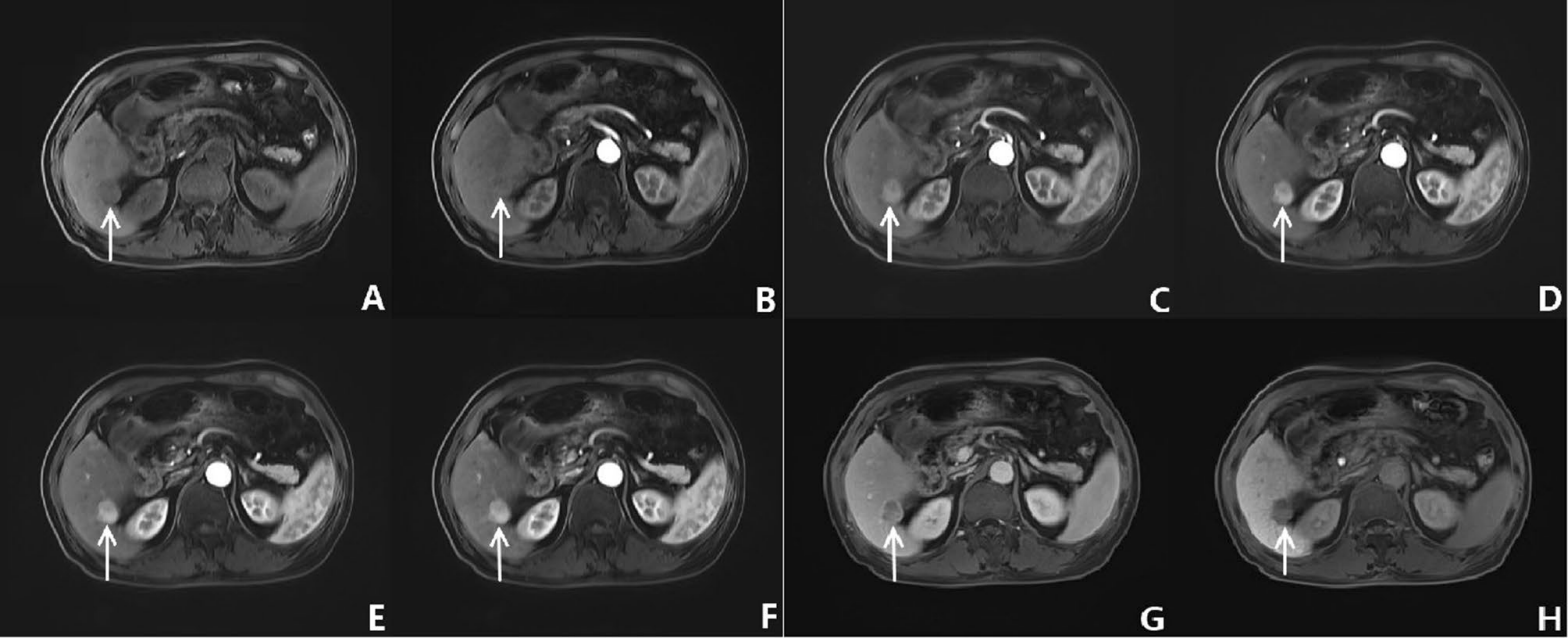
Fifty-eight-year-old man on gadoxetate acid disodium-enhanced liver MR examination using CDT-VIBE sequence. Precontrast phase (**A**), followed by five subphases of CDT-VIBE at (**B**)–(**F**), The portal venous phase (**H**) and hepatobiliary delay period (**G**) were shown by VIBE. MR images showed a 2.4 cm mass (white arrows) in S6/8. This mass presents peripheral arterial enhancement and washout in portal venous phase and hepatobiliary delay period. We proved this mass to be hepatocellular carcinoma by Pathologic examination. The scores of artifact degree showed that (**A**) was 1 point, (**B**) and (**C**) were 3 points, and (**D**)–(**G**) was 1 point. Respiratory movement artifacts were more likely to occur in the early phase of CDT-VIBE enhancement.

Our results show that the motion artifact scores of the five-phase images of the StarVIBE sequence and the CDT-VIBE sequence are similar to those of the conventional single-artery-phase VIBE sequence. In contrast, the best CDT-VIBE images are superior to the two single-artery-phase images. The proportion of severe motion artifacts (motion artifact score of 4) in the scans was much lower with the CDT-VIBE sequence and the StarVIBE sequence than with the VIBE sequence, which may be related to the high temporal resolution of the CDT-VIBE sequence and the k-center overlap filling of the StarVIBE sequence. Since dyspnea in gadoxetic acid disodium enhancement is usually transient. Of short duration, the high temporal resolution of CDT-VIBE 3 s a phase facilitates avoidance of transient dyspnea and provides images with no or mild artifacts by the adjacent phase, so CDT-VIBE can give good quality arterial phase images, although it also shows transient dyspnea. The StarVIBE sequence, on the other hand, has a good suppression of transient respiratory motion artifacts caused by TSM by taking advantage of oversampling in the center of K space and phase encoding in different readout directions. In the CDT-VIBE sequence, the motion artifact scores in phases I and V were significantly higher than in phases III and IV (Figs. 5 and 6). Our results are consistent with previous studies by Park et al.^15^^.^ and Wei et al.^18^, who reported that the most severe motion artifacts appeared in the first sub-stage when they used multiple arterial stages. Furthermore, nearly half of the transient severe motion artifacts appeared in the early multi-arterial phase, which may also explain the strong motion artifacts in the first stage of the multi-arterial phase of CDT-VIBE. The increase in motion artifacts in the fifth stage may be that the optimal breath-hold time for patients is 10 s, making it more challenging to hold the breath. Previous studies found radial streak artifacts in StarVIBE sequences, which may be the reason for the poor artifact scores of StarVIBE sequences compared to CDT-VIBE sequences in this study^20,21^.

We should also acknowledge several limitations of our study. First, the sample size of the current study was relatively small, limiting the ability to draw a firm conclusion. Second, the scanning parameters of the three groups of sequences are not completely matched, which may affect the evaluation of image quality, such as spatial resolution and signal-to-noise ratio; Third, We did not evaluate the diagnostic performance of the three sequences in lesion detection and significance , and further studies are warranted to focus on this issue.

In conclusion, using of multi-arterial acquisition method of gadoxetate disodium enhanced liver magnetic resonance scanning can obtain better late arterial phase and provide high-quality images with fewer motion artifacts, which can be of potential value for specific clinical applications.

## Data Availability

The datasets used and analyzed during the current study are available from the corresponding author on reasonable request.
